# Development and psychometric properties of a short version of the Patient Continuity of Care Questionnaire

**DOI:** 10.1111/hex.13728

**Published:** 2023-02-16

**Authors:** Emma Safstrom, Kristofer Arestedt, Heather D. Hadjistavropoulos, Maria Liljeroos, Lena Nordgren, Tiny Jaarsma, Anna Stromberg

**Affiliations:** ^1^ Nyköping Hospital Sörmland County Council Nyköping Sweden; ^2^ Department of Health, Medicine and Caring Sciences Linköping University Linköping Sweden; ^3^ Center for Clinical Research Sörmland Uppsala University Eskilstuna Sweden; ^4^ Department of Health and Caring Sciences, Faculty of Health and Life Sciences Linnaeus University Kalmar Sweden; ^5^ Department of Research Region Kalmar County Kalmar Sweden; ^6^ Department of Psychology University of Regina Regina Saskatchewan Canada; ^7^ Department of Public Health and Caring Sciences Uppsala University Uppsala Sweden; ^8^ Department of Nursing Science, Julius Center University Medical Center Utrecht Utrecht The Netherlands; ^9^ Department of Cardiology Linköping University Linköping Sweden

**Keywords:** continuity of care, patient discharge, psychometrics, quality of care, reproducibility of results, surveys and questionnaires, validation studies

## Abstract

**Introduction:**

Hospitalization due to cardiac conditions is increasing worldwide, and follow‐up after hospitalization usually occurs in a different healthcare setting than the one providing treatment during hospitalization. This leads to a risk of fragmented care and increases the need for coordination and continuity of care after hospitalization. Furthermore, international reports highlight the importance of improving continuity of care and state that it is an essential indicator of the quality of care. Patients’ perceptions of continuity of care can be evaluated using the Patient Continuity of Care Questionnaire (PCCQ). However, the original version is extensive and may prove burdensome to complete; therefore, we aimed to develop and evaluate a short version of the PCCQ.

**Methods:**

This was a psychometric validation study. Content validity was evaluated among user groups, including patients (*n* = 7), healthcare personnel (*n* = 15), and researchers (*n* = 7). Based on the results of the content validity and conceptual discussions among the authors, 12 items were included in the short version. Data from patients were collected using a consecutive sampling procedure involving patients 6 weeks after hospitalization due to cardiac conditions. Rasch analysis was used to evaluate the psychometric properties of the short version of the PCCQ.

**Results:**

A total of 1000 patients were included [mean age 72 (SD = 10), 66% males]. The PCCQ‐12 presented a satisfactory overall model fit and a person separation index of 0.79 (Cronbach's *α*: .91, ordinal *α*: .94). However, three items presented individual item misfits. No evidence of multidimensionality was found, meaning that a total score can be calculated. A total of four items presented evidence of response dependence but, according to the analysis, this did not seem to affect the measurement properties or reliability of the PCCQ‐12. We found that the first two response options were disordered in all items. However, the reliability remained the same when these response options were amended. In future research, the benefits of the four response options could be evaluated.

**Conclusion:**

The PCCQ‐12 has sound psychometric properties and is ready to be used in clinical and research settings to measure patients' perceptions of continuity of care after hospitalization.

**Patient or Public Contribution:**

Patients, healthcare personnel and researchers were involved in the study because they were invited to select items relevant to the short version of the questionnaire.

## 1 INTRODUCTION

Healthcare utilization due to cardiac conditions is increasing worldwide, with an average of 2400 patients per 100,000 population in Europe discharged from a stay in hospital due to cardiac conditions every year.^1^ The period after discharge is especially critical for patients with cardiac conditions because it includes challenges such as adapting to a new diagnosis and performing self‐care, including handling complex medical regimens, monitoring symptoms and managing deterioration. Comorbidity is common in this patient population, making symptom monitoring and management even more complex.^2^, ^3^, ^4^, ^5^, ^6^ Follow‐up after hospitalization due to cardiac conditions usually occurs with a different healthcare provider than during hospitalization. This poses a risk of fragmented care, thus making coordination and continuity of care even more crucial.^7^ Due to the complexity of the post‐discharge period, the rehospitalization rate within 30 days is an established quality indicator.

Continuity of care is an aspect of transitional care^8^ that can be defined as ‘the extent to which a series of healthcare services is experienced as connected and coherent and is consistent with a patient's health needs and personal circumstances’.^9^ This definition refers to a seamless transition, consistent communication, and care coordination over time and between settings and providers.^10^, ^11^ Three aspects of continuity of care have frequently been described: *Interpersonal continuity* refers to subjective experiences of a caring relationship between patients and healthcare personnel. *Informational continuity* describes the availability and use of clinical and psychosocial information from previous events to provide adequate care for the patient.^9^, ^12^, ^13^ Informational continuity also includes information transferring to patients regarding health conditions and what to expect after discharge. Receiving this information is a way for patients to experience continuity.^9^ *Management continuity* means effective collaboration across different healthcare settings to provide seamless care. Patients experience management continuity when the care plan is clearly described, including scheduled follow‐ups and planned exams or future medical treatment.^9^, ^12^, ^13^ A recent conceptual analysis emphasized that continuity of care only occurs when all three aspects are aligned and integrated.^14^

Modern healthcare systems are increasingly complex and divided into numerous specialities with different healthcare providers, which impedes continuity of care.^4^, ^11^, ^15^ Flaws in continuity and insufficiently coordinated care during the transition from inpatient to outpatient healthcare may result in fragmented care, and this has been found to jeopardize both the quality of care and patient safety.^4^, ^7^, ^16^, ^17^ Furthermore, patients with cardiac conditions have described the discharge process and clinical care pathways as fragmented and difficult to understand.^18^ Several reports, including a recent summary from the World Health Organization, highlight that enhanced continuity of care is critical for improving patient outcomes and quality of care.^19^, ^20^ Continuity of care has been found to improve clinical outcomes such as health‐related quality of life and reduce rehospitalizations.^21^, ^22^ However, even though continuity is a healthcare priority, there is a lack of short, validated questionnaires to measure patients' perceptions of this aspect of care. If such questionnaires were available, the results could guide quality improvement initiatives and help to evaluate the continuity of care interventions.^23^, ^24^

There are different objective measures of continuity of care, such as the Continuity of Care Index or the Usual Provider Index^25^ but they do not include the patients' perceptions of continuity. In addition, most available patient‐reported questionnaires do not address the posthospital period.^26^, ^27^, ^28^ However, Hadjistavropoulos and colleagues developed the Patient Continuity of Care Questionnaire (PCCQ), which involves patients' perceptions of the different aspects of continuity of care and addresses the period immediately following hospital discharge. In the original version of PCCQ, 27 items form six subscales, meaning that no total score is available.^29^

In a prior study, we translated and culturally adapted the PCCQ from English into Swedish and confirmed the proposed factor structure.^30^ However, during translation and think‐aloud interviews, that completing the PCCQ was found time‐consuming. Furthermore, when it came to items regarding mainly community care, as many as 50% of patients had responded ‘not applicable’. When the factor structure was analysed, evidence was found that the number of items could be further reduced and that it could be possible to use the PCCQ as a unidimensional measure and calculate a total score.^30^

A short version is advantageous because it only includes the most essential items without compromising the questionnaire's ability to take adequate measurements. In addition, answering fewer items would reduce respondents' burden and be time‐saving for clinicians who work in time‐restricted settings. Moreover, a total score makes comparing results between diagnoses or interventions easier. Therefore, this study aimed to develop a shorter version of the PCCQ with items focusing on continuity of medical care in the context of the acute hospitalization and discharge period for patients hospitalized due to cardiac conditions. Furthermore, we aimed to evaluate the psychometric properties, response dependence, and dimensionality of this short version of the PCCQ using the Rasch measurement model.

## 2 METHOD

### 2.1 Study design

This was a psychometric evaluation study.

### 2.2 Setting and participants

To evaluate the psychometric properties of the short version of the PCCQ, we used data already collected (*n* = 725) for prior validation of the full instrument,^30^ and an additional 275 patients from the same data collection were included in this study. This was a multicenter study, and data were collected from patients discharged after hospitalization due to cardiac conditions from four different hospitals (one university hospital, two county hospitals, and one district hospital) in two counties in the centre of Sweden. Patients were included using consecutive inclusion procedures and were eligible if they had been hospitalized due to angina (ICD: I20.0, I20.1, I20.8, I20.9), myocardial infarction (ICD: I21.0, I21.1, I21.2, I21.3, I21.4, I21.9), atrial fibrillation (ICD: I48.0, I48.1, I48.2, I48.9) or heart failure (ICD: I42.0, I50.0, I50.1, I50.9). Further criteria for inclusion were age ≥18 years and being hospitalized >24 h. The exclusion criteria were dementia with severe cognitive decline; inability to read or understand Swedish; expected survival of <3 months; discharge to a nursing home; or being resident in another county.

The first author and research nurses scrutinized lists of patients discharged due to angina, myocardial infarction, atrial fibrillation, or heart failure provided by hospital administrators. Eligible patients were contacted by regular mail and received study information, a written informed consent form, the PCCQ, and questions about patient demographics within 4–6 weeks after discharge. In addition, a reminder was sent to patients who had not responded after 4–5 weeks.

### 2.3 The PCCQ

The PCCQ is a generic questionnaire addressing patients' perceptions of continuity of care during hospitalization and after discharge. This includes items regarding both medical and community care. The instructions on the questionnaire state that the items are designed to assess the care received around the time of discharge from the hospital. The first part of the questionnaire addresses the period before discharge, and the second part addresses the period after discharge. Five response options range from ‘strongly disagree’ to ‘strongly agree’, scored from 1 to 5, with higher scores indicating better continuity of care. All items also have a ‘not applicable’ option. The original PCCQ has six subscales: (1) information transfer to patients, (2) relationships with providers in the hospital, (3) relationships with providers in the community, (4) management of written forms, (5) management of follow‐up, and (6) management of communication among providers.^29^

### 2.4 Selecting items for the short version of the PCCQ

User‐group representatives (researchers, patients, and healthcare personnel) were chosen via a convenience sampling procedure. They were invited to evaluate the content validity and suggest items for the short version.

Seven researchers were contacted by e‐mail and asked to rate the relevance of the items. The researchers (six women, one man, age range 42–64 years) with expertise in the continuity of care and instrument development rated each item on a 4‐point scale, ranging from not relevant to highly relevant. Based on their ratings, the content validity index was calculated at an item level. Considering the number of researchers, the recommended cut‐off value was 0.78.^31^ In addition, patients were asked to mark 10 items they considered most relevant in relation to Haggerty's definition of continuity of care.^9^ Seven patients (three women, four men) had all previously been hospitalized due to cardiac conditions. The 15 healthcare personnel (14 women, 1 man, age range 39–63 years) were nurses and physicians working on hospital wards, in outpatient care clinics, primary care clinics, and as discharge coordinators (nurses) working on hospital wards. The results were compiled, and if more than one‐third of the patients or healthcare personnel found an item to be relevant, that item was considered relevant to those user groups (Supporting Information: Table S1).

Thereafter, items to be included in the short version were selected after consensus was reached within the group of authors. This consensus was preceded by conceptually based discussions among the authors as well as scrutiny of the concept of continuity of care and the feedback from user groups on the content validity. Two of the items regarding informational continuity were not rated as relevant by any user groups and were not included in the short version: information regarding nonacute symptoms and information regarding activities. Eight of the items originally regarding relational continuity were not included in the short version: Five of these address satisfaction with care rather than continuity of care. In addition, three items (healthcare personnel understood expectations, confidence in healthcare personnel, and healthcare personnel communicated well with each other) were not rated as relevant by any user groups and were not included in the short version. Five items regarding management continuity were not included in the short version. The items concerned information transfer between healthcare personnel and settings. Prior studies have found that patients assume that communication between healthcare personnel and settings works as planned until proven otherwise. A qualitative meta‐summary by Haggerty and colleagues describes that, as long as no problems have occurred, patients presume that healthcare personnel communicate and retrieve relevant information and have an agreed‐upon care plan.^9^

Based on the results of the content validity and discussion about the concept, the authors finally selected 12 items that were deemed to include the concept of continuity of care: Four items regarding informational continuity, four items regarding relational continuity, and four items regarding management continuity. Items from every subscale except subscale six (the management of forms) are represented in the short version. Detailed information about the reasons for exclusion for each item is presented in Supporting Information: Table S1. The original developer of the scale (Dr Hadjistavropoulos) was consulted during all the process steps and co‐authored the paper.

### 2.5 Analysis

Descriptive statistics were used to describe patient characteristics and data quality. The psychometric properties of the 12 items in the proposed short version of the PCCQ were evaluated with the polytomous Rasch model, using RUMM2030 software. The Rasch model is often regarded as an item response theory model. The response option ‘not applicable’ was coded as ‘missing’ for the analysis. The Rasch model does not require complete data but can handle missing values.^32^ Cronbach's *α* and ordinal *α* were estimated using R V.3.5.1, psych package.^33^

#### 2.5.1 Rach analysis

##### Overall fit and individual item fit

The mean and standard deviation (SD) of items and person residual values were estimated to examine the overall fit between the data and the model. The data fits the model when standardized residuals are normally distributed, that is, when the mean is close to 0, and the SD is close to 1. Furthermore, the *Χ* ^2^‐based item‐trait interaction statistic was expected to be nonsignificant.^34^, ^35^

The fit of individual items was evaluated by estimating the standardized residuals, *Χ* ^2^ values, and item characteristic curves. An item was considered to fit the model if the standardized residuals for the item lay in the range −2.5 < *x* < +2.5^35^, ^36^ and the *Χ* ^2^ test was nonsignificant.^34^ Because multiple tests were conducted, the *p*‐values were Bonferroni corrected at 0.004.^37^

##### Targeting

Targeting refers to the alignment of person and item‐thresholds locations. Therefore, the mean value of a person's location should be close to the mean value of the item (i.e., 0 logits). Item thresholds are expected to cover about the same range of the logit scale as the person locations.^38^

##### Local independence

Response dependence is when the response to one item depends on the response to another. The correlation of the residuals between two items is estimated to find signs of response dependence. If that correlation is greater than 0.2 above the average of all item residual correlations, it is considered a sign of response dependence.^39^ Response dependency can affect the measurement properties of an instrument and cause the reliability to be falsely elevated.^40^ Whether the response dependency has led to any measurement bias or falsely elevated reliability can be evaluated: The response‐dependent items are combined into a subtest item, and after that, the measurement properties and reliability of the model before and after the subtest item are compared.^41^

##### Dimensionality

The Rasch measurement model assumes that the items represent only one dimension. Therefore, a poor model fit can indicate multidimensionality; however, some analyses can be made to evaluate dimensionality in more detail. Principal component analysis (PCA) on item residuals followed by a series of *t*‐tests was estimated to further explore dimensionality. Person locations derived from items with positive residual loading (≥+0.3) on the first component were compared to those with negative residual loading (<−0.3). The *t*‐tests then explore whether there are any significant differences between the person location with positive loading versus the person location with negative loading. Unidimensionality is supported if fewer than 5% of the *t*‐tests are significant (*p* < .05) or if the lower bound of the Agresti–Coull binominal 95% confidence interval (CI) overlaps or exceeds 5%.^42^, ^43^

##### Person Separation Index

Reliability was evaluated using the Person Separation Index, which is interpreted in the same way as Cronbach's *α* and reflects the ability to differentiate between people at different person locations, that is, the instruments' ability to make accurate measurements. A minimum value of 0.7 is recommended.^42^ To enable comparison, Cronbach's *α* and ordinal *α* ^44^, ^45^ were also calculated.

##### Differential Item Functioning (DIF)

DIF was analysed in terms of age and gender to evaluate invariance between groups, for example, if the PCCQ provides accurate measures regardless of whether a woman or a man or a younger or older patient responded. To evaluate signs of DIF, the sample is divided into groups based on gender or age. After that, a two‐way ANOVA with Bonferroni corrected *p* values is estimated. In addition, item characteristic curves are inspected. Uniform DIF can imply the presence of group bias (e.g., that the measures provided by the instrument are affected by age or gender).^46^ Age was dichotomized based on the included sample's median value into <73 years and ≥73 years.

##### Category thresholds

One requirement of the Rasch model is that the response options are ranged in an ordered set along the continuum of the latent construct, that is, that the first response option is the easiest, that the second response option is the second easiest, and so forth. When the response options are in order, this implies that they are working as expected, strengthening the construct validity. The thresholds were evaluated by analysing the category probability curves and the category thresholds. If reversed thresholds are found, the response options can be amended, whereafter the model is reevaluated. If there are only minor differences between the original and the reevaluated model, the interpretation is that the disordered response options do not affect the instrument's measurement properties.^47^, ^48^

#### 2.5.2 Reproducibility

A linear regression analysis was conducted to evaluate reproducibility, that is, to assess the amount of variance in the original version of the PCCQ explained by the short version. The explained variance was assessed using the *R* ^2^ value. A *p* value of .05 was considered significant. A linear regression analysis was conducted using IBM SPSS Statistics 25.

## 3 RESULTS

### 3.1 Sample

A total of 1000 patients were included (66% males, mean age 72 years, SD = 10). The diagnoses at hospitalization were acute myocardial infarction 36% (*n* = 359), atrial fibrillation 26% (*n* = 255), angina 21% (*n* = 210), and heart failure 17% (*n* = 176).

### 3.2 Item statistics

All response options were used for the items included in the PCCQ‐12, and the percentage of internal missing data ranged from 2% to 4% (Table 1). The mean raw total score for the PCCQ‐12 was 44.6 (SD = 11.6); 11 patients (1.1%) scored the lowest possible score, and 52 patients (5.2%) scored 60, the highest possible score.

**Table 1:** Data quality for the 12 items selected for the short version of the PCCQ‐12 (*n* = 1000).

		Response options						
Item description	Type of continuity	Strongly disagree	Somewhat disagree	Neither agree nor disagree	Somewhat agree	Strongly agree	Not applicable	Missing data
I was provided with clear information on my diagnosis.	Informational	1%	1%	6%	20%	69%	1%	2%
I was provided with clear information on my prognosis.	Informational	4%	4%	15%	26%	43%	4%	4%
I was given information on symptoms that may signal a need to seek urgent medical attention & whom to contact for these symptoms (e.g., specialist, family physician, homecare).	Informational	6%	4%	14%	24%	45%	5%	3%
I was given complete information on my medications (e.g., type, purpose, how given, when, how often for how long, how much, side effects, drug interactions, nature and frequency of blood work).	Informational	3%	4%	10%	29%	49%	2%	3%
I was given information on follow‐up appointments that have been made for me and appointments I have to schedule for myself.	Management	5%	4%	9%	22%	55%	3%	2%
I was informed of ongoing treatment that may be required after discharge (e.g., purpose, how, when) and whether I will have ongoing contact with providers of my care (e.g., physician, etc.).	Management	14%	5%	16%	19%	26%	17%	3%
I felt ‘known’ (e.g., current clinical condition and events) by the providers involved in my care.	Relational	2%	3%	10%	24%	56%	3%	2%
A well‐developed and realistic follow‐up plan was prepared and explained to me.	Management	9%	5%	17%	25%	33%	7%	4%
I felt adequately prepared for discharge.	Relational	4%	4%	11%	22%	55%	2%	2%
I feel ‘known’ (e.g., current health condition) by my present providers who have taken over my care since discharge.	Relational	4%	3%	14%	20%	43%	13%	4%
I have confidence in my providers who have taken over my care since discharge.	Relational	4%	3%	13%	19%	45%	12%	4%
I was given consistent information by all providers about my care.	Management	9%	5%	21%	18%	33%	10%	4%

Abbreviation: PCCQ, Patient Continuity of Care Questionnaire.

### 3.3 Rasch analysis

**Figure 1:**
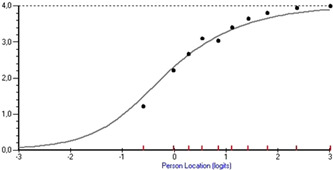
Displaying the item characteristic curve for item confidence in responsible healthcare personnel after discharge, the item that deviated significantly from the model. The line represents the model, and the dots represent class intervals of 10 persons per group, revealing only minor deviations between the model and the persons.

The overall fit of the PCCQ‐12 to the Rasch model was 182.91 (*p* < .001), a significance that is to be expected due to the sample size. Two items were just outside the expected range of the fit residual values but did not deviate statistically from the model: information about treatment after discharge and healthcare personnel in hospital had knowledge of the medical situation. In contrast, the item regarding confidence in responsible healthcare personnel after discharge deviated significantly from the model, with a significant *Χ* ^2^ value and a fit residual value of −3.490. However, an inspection of the item characteristic curve for this item revealed only minor deviations (Figure 1).

**Figure 2:**
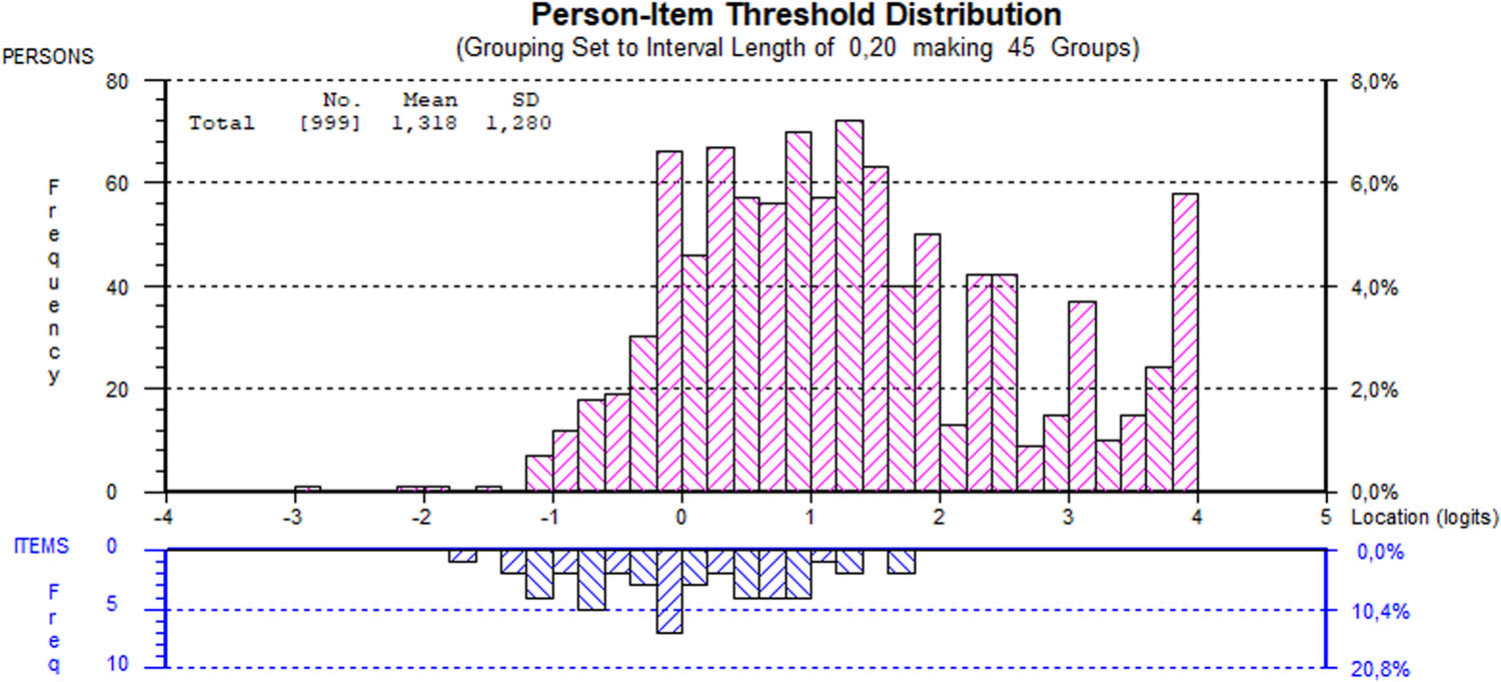
Targeting for persons and items. Distribution of the locations of persons (top histogram) and the PCCQ‐12 category thresholds (bottom histogram) on the common latent continuity of care variable. The figure reveals that the persons seem to experience better continuity of care than the items in the PCCQ‐12 can measure. The gaps imply a lack of items to measure the best continuity of care (for patients located above two logits on the scale). PCCQ, Patient Continuity of Care Questionnaire.

The targeting is presented in Figure 2 and reveals that the items constructing the PCCQ‐12 cover a large proportion of the patients' perceived continuity of care, except for those individuals who experienced the best continuity of care. In addition, the mean person location relative to the items was 1.32 (SD = 1.28), further indicating that patients tend to experience better continuity than the items can measure.

The person‐item residual correlation matrix was estimated to evaluate any signs of local dependency. Two pairs of items presented a correlation of 0.2 above the medium correlation: 'information on diagnosis' and 'information on prognosis' was the first pair of items that showed signs of response dependency. The second pair of items were 'feeling known by healthcare personnel after discharge' and 'having confidence in healthcare personnel after discharge'. Each of these two pairs of items was combined into a larger subtest item to evaluate whether the response dependency had an impact on the measurement properties of the PCCQ‐12, whereafter model fit and PSI were evaluated. As presented in Table 2, the change in person location, person fit, and PSI when dealing with the response dependence problem was negligible.

**Table 2:** Changes in person location, person fit and person separation index in different models when considering local dependency problems.

	Model 1^a^	Model 2^b^	Difference between Models 1 and 2	Model 3^c^	Difference between Models 1 and 3	Model 4^d^	Difference between Models 1 and 4
Mean person location	1.317	1.241	0.076	1.241	0.076	1.151	0.166
Mean person fit residual	−0.297	−0.311	0.014	−0.294	−0.003	−0.315	0.018
PSI	0.786	0.777	0.009	0.768	0.018	0.758	0.028

Abbreviation: PSI, Person Separation Index.

^a^ Model 1: Includes 12 individual items.

^b^ Model 2: The items regarding information on diagnosis and information on prognosis are combined into a ‘subtest’ item.

^c^ Model 3: The items concerning feeling known by healthcare personnel after discharge and having confidence in healthcare personnel after discharge are combined into a ‘subtest’ item.

^d^ Model 4: Both pairs of items combined into a subtest item.

The (PCA)/*t*‐test was estimated to evaluate any signs of multidimensionality. Person locations derived from items with positive residual loading (≥+0.3) on the first component were compared to those with negative residual loading (<−0.3) with a *t*‐test. A total of 3.7% was significant, and the Agresti–Coull binominal 95% CI was 0.03–0.05, thus indicating that the PCCQ‐12 is unidimensional.

Reliability (PSI) was 0.79, indicating that the PCCQ‐12 can distinguish between 2.9 distinct groups of individuals. The Cronbach's *α* was .91 (95% CI: 0.90–0.91), and ordinal *α* was .94 (95% CI: 0.87–0.98).

No DIF was found for age, but nonuniform DIF for gender was found for the item concerning being prepared for discharge (*F*(1, 9) = 3.17, *p* < .001); however, there was no clear interaction, and when we reran the analysis reducing the number of the class interval from 10 to 8, no signs of DIF were found. This indicates that DIF does not affect the measurement properties of the PCCQ‐12.

At this point, we evaluated the category thresholds. We found disordered thresholds for all items (Table 3) located at the first two response options, ‘strongly disagree’ and ‘somewhat disagree,’ which was further confirmed by scrutinizing the category probabilistic curves. Consequently, the response options were rescored by collapsing the first two. Rescoring the response options resulted in ordered response category thresholds for all items, while the mean person location decreased only slightly from 1.317 to 1.132, and PSI remained at 0.79. Furthermore, following the amendment of the response categories, the individual item fit residual improved for the misfitting items (information about treatment after discharge, healthcare personnel in the hospital had knowledge of the medical situation, and having confidence in responsible healthcare personnel after discharge). Moreover, amending the first and second response options did not alter the results regarding response dependence, dimensionality, targeting, or DIF.

**Table 3:** Individual item‐fit statistics and thresholds for the items in the PCCQ‐12 (*n* = 1000).

Item	Location	SE	Fit residual	*Χ* ^2^	*p* Value^a^	Thresholds			
						0 → 1	1 → 2	2 → 3	3 → 4
Information on diagnosis	−0.770	0.051	0.299	15.90	.069	−0.310	−1.715	−1.029	−0.025
Information on prognosis	0.008	0.041	1.256	15.79	.072	−0.173	−1.042	0.131	1.115
Information on acute symptoms	0.127	0.038	2.369	15.54	.077	0.277	−0.779	0.078	0.934
Information on medication	−0.179	0.042	1.503	12.26	.199	−0.465	−0.780	−0.453	0.984
Information on follow‐up appointments	−0.175	0.040	−0.708	16.34	.060	−0.086	−0.809	−0.314	0.509
Information on ongoing treatment	0.748	0.037	2.601	10.67	.299	1.220	−0.611	0.706	1.678
HCP knew about situation	−0.445	0.045	−0.956	14.94	.093	−0.924	−1.136	−0.326	0.608
Follow‐up plan explained	0.470	0.038	−1.504	18.24	.033	0.536	−0.749	0.413	1.680
Sufficiently prepared for discharge	−0.099	0.039	2.443	12.47	.188	−0.055	−0.780	−0.146	0.587
Felt known by HCP after discharge	−0.062	0.044	−2.773	22.47	.008	−0.108	−1.281	0.230	0.910
Confidence in HCP after discharge	−0.122	0.044	−3.490	31.26	<.001	−0.111	−1.240	0.160	0.703
Consistent information	0.498	0.038	1.462	15.02	.091	0.737	−1.045	0.970	1.329

Abbreviations: HCP, Healthcare Personnel; PCCQ, Patient Continuity of Care Questionnaire; SE, standard error.

^a^ Bonferroni corrected 0.004.

### 3.4 Reproducibility

According to the linear regression model, the PCCQ‐12 explained 85.9% of the total variance in the original version (*F*(1, 998) = 758.43, *p* < .001).

## 4 DISCUSSION

We developed and evaluated a 12‐item short version of the PCCQ (the PCCQ‐12). We did so by considering the content validity feedback from user groups and conceptual discussions among the authors regarding the definition and concept of continuity of care. When the Rasch model was applied, the PCCQ‐12 was found to have sound psychometric properties and good reliability. In addition, the analysis supported the possibility of calculating an interpretable total score. We found some response dependence and disordered thresholds; however, none of these had a significant impact on the questionnaire's measurement properties.

During content validity evaluation and discussion among the authors, the number of items was reduced from 27 to 12. The selection of items must be undertaken with conceptual considerations in mind to ensure that important aspects of the latent trait are not lost.^49^ To ensure that items were selected according to conceptual considerations, retaining relevant aspects of continuity of care, we included user groups in the process: researchers, healthcare personnel, and patients, and the selection of items for the short version was guided by their feedback. To include a broad perspective, we included healthcare personnel from different settings—hospital wards, outpatient clinics and primary care. Seven patients with experience of being hospitalized due to a cardiac condition were also included in the validation. The psychometric properties of the items were evaluated using Rasch analysis. Rasch can be used as a guide to which items to include when developing an instrument, but can also be used, as we did, to evaluate the measurement functioning of a set of items that are conceptually relevant to form a latent variable.^50^

Even though the number of items was reduced from 27 to 12, we argue that the short version can still be considered a relevant measure of patients' perception of continuity of care. The PCCQ‐12 includes four items on relational continuity, four on informational continuity, and four on management continuity.

In total, two items regarding informational continuity were not included in the short version: The item concerning information on nonacute symptoms was not included because it was not rated as relevant by any user group. Initially, we were hesitant to not include this item but argued that the item on information regarding acute symptoms was more relevant, considering that the PCCQ addresses the initial posthospital period. This period after discharge is described as particularly vulnerable with a high risk of adverse events.^51^ Patients have stressed that it is important, in cooperation with their healthcare personnel, for them to make a plan for how to handle adverse events.^9^ Meanwhile, we found the item regarding information and advice on activities more related to self‐care than to continuity of care,^52^ and it was not included in the short version. Thus, the PCCQ‐12 includes four items regarding informational continuity which address information on diagnosis, prognosis, medication, and how to handle acute symptoms. These have been identified in prior research as key factors for patients to perceive continuity of care after discharge.^9^, ^53^, ^54^

Regarding relational continuity, eight items were not included in the short version. Five of these explicitly address patient satisfaction. We argue that even though satisfaction is related to continuity of care^10^ and the literature describes that satisfaction is associated with continuity of care and a possible outcome of continuity of care,^55^, ^56^, ^57^, ^58^, ^59^ satisfaction with care is not equivalent to continuity of care. Since this study aimed to develop a short version focusing on continuity of care after hospitalization, we decided to not include items that explicitly measure patients' satisfaction. Thus, the PCCQ‐12 includes four items on relational continuity, which address feeling prepared for discharge, feeling known by healthcare personnel before discharge, and feeling known by and having confidence in healthcare personnel after discharge. We were initially surprised that none of the user groups rated ‘healthcare personnel understood my expectations’ or ‘confidence in healthcare personnel before discharge’ as important items to include in the short version. However, considering that the PCCQ measures perceptions of continuity of care during the period before *and after* hospitalization, it is understandable that the item ‘feeling known by healthcare personnel after hospitalization’ and ‘having confidence in healthcare personnel after discharge’ were valued more highly by the user groups. Therefore, we considered these items more essential in relation to continuity of care after discharge. This is also supported by prior research: Two recent qualitative studies recognize the importance of having confidence in healthcare personnel and feeling known after discharge. Griffiths and colleagues^53^ found that the general practitioner played an important role for the patient after discharge in explaining the new diagnosis, reviewing new medications, reviewing vitals, and discussing the plan for further treatment or follow‐up. Patients relied on their general practitioner after discharge and felt that the post‐discharge visit was essential for coordinating care and maintaining continuity of care. Moreover, Perrault‐Sequeira and colleagues^60^ found that patients perceived the follow‐up with their general practitioner after hospitalization to be essential in helping them navigate the changes in their health and support during their transition from hospital to home.

A total of five items regarding management continuity were not included in the short version. In three of the items, patients were asked whether any forms were lost during discharge, and two of them addressed how the healthcare personnel in the hospital communicated with the general practitioner and community care representatives. We argue that patients are unaware of any miscommunication between healthcare settings and assume that everything has gone smoothly until proven otherwise,^9^ and therefore these items were not included in the short version. However, Griffiths et al.^53^ and Perrault‐Sequeira et al.^60^ found that patients did notice if information, such as a discharge summary, had been transferred from the hospital to their general practitioner. Nevertheless, they felt that the discharge summary did not cover all of the important facts regarding their care. In summary, the PCCQ‐12 includes four items on management continuity. Two of them address how well‐informed patients are about planned follow‐up and ongoing treatment after discharge. Prior research has shown major shortcomings within this area, with patients hospitalized due to heart failure experiencing a lack of information about medicines and planned follow‐up.^61^ Therefore, since information regarding follow‐up appointments is essential for continuity after hospitalization, we decided to include the item concerning information on follow‐up appointments in the short version even though 17% of the patients answered ‘not applicable’ to this item. Furthermore, having the follow‐up plan explained has been described by patients in a recent qualitative study as the key feature during the posthospital period.^53^ Therefore, we decided to include this item in the short version. Finally, it is noteworthy that items regarding management continuity received the lowest scoring by the patients, since the proportion of patients who answered ‘strongly disagree’ or ‘somewhat disagree’ ranged between 14% and 19% in three of the items.

The Rasch analysis supported the claim that the PCCQ‐12 is a unidimensional instrument. Since items representing all three types of continuity are included in the short version, the PCCQ‐12 conceptually covers all three types of continuity. Informational, relational, and management continuity have been referred to as dimensions of continuity of care in the literature; however, Haggerty and colleagues have expressed discomfort with using the term dimensions. Instead, they argue that the components of continuity are parallel and intertwined with each other and cannot be separated. All three types of continuity are needed for patients to perceive continuity of care. Moreover, all three types exist in all settings, even though they might have different degrees of importance depending on the patient's situation or the care context.^12^, ^13^ When reducing the number of items, the two items representing community care were not included in the PCCQ‐12. Hence, the PCCQ‐12 is mainly suitable for evaluating medical healthcare continuity after acute care hospitalization, not community care. However, the original version of the PCCQ is recommended for patients with extensive needs for community care. The two versions allow healthcare personnel and researchers to use the questionnaire that best suits their purpose. For both research and quality improvement projects in a clinical setting, it is a great asset to have the opportunity to choose between these two questionnaires: the original version, including community care, and the PCCQ‐12, which focuses on medical care.

Of the 12 items in the short version, three items presented misfits with residuals of a magnitude greater than ±2.5, which could be caused by multidimensionality or response dependence. Our analysis found no signs of multidimensionality, but two pairs of items did present signs of response dependence. When we scrutinized the item content, it seems likely that if a patient is informed of their prognosis, they are also informed about the diagnosis. Moreover, if a patient feels that the healthcare personnel know about their medical situation, they will have confidence in that healthcare personnel. Despite this, when analysing the response dependence further, we found that it did not have a significant impact on the measurement properties of the PCCQ‐12.

Since the PCCQ‐12 is unidimensional, a total score can be found. When calculating the total score, response options 1–5 should be used, and the response option ‘not applicable’ should be treated as a missing response and not scored. Consequently, the total score of the PCCQ‐12 has a potential range from 12 to 60.

Any future validations of the PCCQ‐12 should evaluate a version where the number of response options is reduced. One advantage of Rasch analysis is its ability to evaluate the function of the response options. We found that the options ‘Strongly disagree’ and ‘Somewhat disagree’ were disordered in all items. This disordering is probably because only a few patients gave these responses or because patients had problems differentiating between these responses. The problematic thresholds were resolved by collapsing the first two response options into one,^47^, ^48^ whereafter, the individual item fit residuals improved. Notably, the response options were amended only for psychometric analyses in this study, and they should not be collapsed for everyday use until a version of the questionnaire using four response options has been validated.

Even though the sample in this study consisted of patients with cardiac conditions, we argue that the PCCQ‐12 can be considered a generic questionnaire since the original PCCQ was developed as a generic questionnaire in medical and orthopaedic care^29^ and has been validated in patients with chronic conditions.^62^, ^63^ Furthermore, in this study, the psychometric properties were evaluated using Rasch analysis, a method that is not sample dependent to the same extent as classical test theory. However, future studies should examine the PCCQ‐12 for DIF in different patient groups to determine whether the instrument can make invariant measurements between different diagnoses.

The PCCQ‐12 can be used in research and clinical practice to evaluate and improve the continuity of care after hospitalization. For example, at a group level, hospital wards and outpatient clinics can use it to evaluate the process of discharge and follow‐up to find areas of insufficient continuity of care that could be improved to enhance the quality of care or used to evaluate interventions aiming to improve continuity. In research, the PCCQ‐12 can evaluate and compare the continuity of care between different diagnoses, sexes, and age groups or evaluate the continuity of care before and after quality improvement interventions.

### 4.1 Limitations

There are some limitations. First, no sample size calculation was made for this study. However, one guideline states that between 10 and 20 people for every threshold in the item set should be adequate to conduct the tests of fit (thus, the proper sample size for the PCCQ‐12 is between 480 and 960 people). The larger the sample size, the greater the power of detecting misfits.^64^ An additional limitation is that only seven patients participated in selecting items for the short version, which might have influenced which items were graded as relevant. However, all of these patients had experienced being hospitalized due to cardiac conditions making them experts within the field. Moreover, we considered the patients' feedback as important as the feedback from healthcare personnel and researchers. Another possible limitation is that the data collection took place 4–5 weeks after discharge, and some patients might not have had their follow‐up appointments at that point. This could have affected their responses to items regarding the period after discharge. However, if data had been collected too long after the hospitalization, there is a risk of recall bias.

We have argued that the PCCQ‐12 correlates to the original PCCQ, and using the Rasch model, we have been able to evaluate its psychometric properties, the homogeneity of the items, and dimensionality. Furthermore, based on our prior knowledge of the continuity of care and numerous conceptual discussions among the authors, we hypothesize that the PCCQ‐12 does measure continuity of care. However, the Rasch model does not allow for the evaluation of construct validity in terms of convergent validity or criterion validity; therefore, the validity of the PCCQ‐12 should be evaluated in future studies.

## 5 CONCLUSION

The PCCQ‐12 is a psychometrically sound questionnaire that is ready to measure patients' perceptions of continuity of care after hospitalization. The overall results of the Rasch analysis indicate that the PCCQ‐12 captures one meaningful common construct and that a total score can be calculated, which is advantageous for comparing individuals or groups. The PCCQ‐12 can be used for research and clinical practice to find areas of insufficient continuity of care that could be improved to enhance the quality of care.

## CONFLICTS OF INTEREST STATEMENT

The authors declare no conflicts of interest.

## ETHICS STATEMENT

This study was conducted in line with the principles of the Declaration of Helsinki. Accordingly, approval was granted by the Regional Ethical Review Board in central Sweden (No. 2017‐226‐31, 2017/610‐32). Informed consent was obtained from all individual participants included in the study.

## Supporting information

Supporting Information.

## Data Availability

Data are available upon reasonable request.
