# Supplementary material for: Development and psychometric properties of a short version of the Patient Continuity of Care Questionnaire
Source: Health Expect. 2023 Feb 16;26(3):1137–48. doi: 10.1111/hex.13728 (PMC10154813; doi:10.1111/hex.13728)
Supplement: Supplementary file 1 — Supporting Information. [file HEX-26--s001.pdf]

Supplementary file. Developing and evaluating the PCCQ-12.

Table S1. Results from content evaluation by user-group representatives and the author's decision on which items to include in the short version of the PCCQ.

	Rated as important by				
	**Researchers (n=7)**	**HCP (n=15)**	**Patients (n=7)**	**Proportion of user groups that found the item relevant**	**Author's decision and reason**
**Information about diagnosis**	No	x	x	2/3	Conceptual relevant.
**Information about prognosis**	No	No	x	1/3	Conceptual relevant.
Information about non-acute symptoms	No	No	No	0/3	Not relevant to user groups.
**Information about acute symptoms**	No	x	x	2/3	Conceptual relevant.
**Information about medication**	No	x	x	2/3	Conceptual relevant.
Information on activities, exercises	No	No	No	0/3	Not relevant to user groups
**Information on follow-up appointments**	x	No	x	2/3	Conceptual relevant.
**Information on ongoing treatment**	x	No	No	1/3	Conceptual relevant.
Understood expectations	No	No	No	0/3	Not relevant to user groups
**HCP knew about situation and medical condition**	x	No	x	2/3	Conceptual relevant.
Confidence in HCP before discharge	No	No	No	0/3	Not relevant to user-groups
Satisfied with information	No	No	No	0/3	Not relevant to user groups. Refers to the concept satisfaction.
Satisfied with emotional support	No	No	No	0/3	Not relevant to user groups. Refers to the concept satisfaction.
Satisfied with opportunities to ask questions and talk	No	No	No	0/3	Not relevant to user groups. Refers to the concept satisfaction.
HCP communicated well with each other	No	No	No	0/3	Not relevant to user groups.
**Follow-up plan explained**	x	x	No	2/3	Conceptual relevant.
**Felt sufficiently prepared for discharge**	x	No	No	1/3	Conceptual relevant.
**Felt known by HCP after discharge**	x	x	No	2/3	Conceptual relevant.
**Confidence in HCP after discharge**	x	No	x	2/3	Conceptual relevant.
Satisfied with information after discharge	x	No	No	1/3	Not relevant to user groups. Refers to the concept satisfaction.
Satisfied with opportunities to ask questions after discharge	No	No	No	0/3	Not relevant to user groups. Refers to the concept satisfaction.

	Rated as important by				
	Researchers (n=7)	HCP (n=15)	Patients (n=7)	Proportion of user groups that found the item relevant	Author's decision and reason
Hospital communicated well with municipality	No	No	No	0/3	Not relevant to user groups. Patients assume that communication between settings work until proved otherwise.
Information transfer from hospital to follow-up care	No	No	No	0/3	Not relevant to user groups. Patients assume that communication between settings work until proved otherwise.
**Consistent information from all HCP**	x	No	No	1/3	Conceptual relevant.
Required forms were filled in	No	No	No	0/3	Not relevant to user groups. Patients assume that communication between settings work until proved otherwise
Required forms were sent	No	No	No	0/3	Not relevant to user groups. Patients assume that communication between settings work until proved otherwise
No forms went missing at discharge	No	No	No	0/3	Not relevant to user groups. Patients assume that communication between settings work until proved otherwise

An 'x' marks when an item scored >0.78 on I-CVI, and when 1/3 of the HCP or patients found the item relevant.

Items included in the PCCQ-12 appears in bold.

HCP; Health Care Personnel, I-CVI; Item level content validity index.
